# 1446. Multidrug-Resistant *Acinetobacter baumannii complex* Cluster Outbreak Investigation

**DOI:** 10.1093/ofid/ofad500.1283

**Published:** 2023-11-27

**Authors:** Divya Chandramohan, Delvina Ford, Erica Beck, Theresa Carroll, Sean O’Neil, Jose Cadena

**Affiliations:** UT Health-San Antonio, San Antonio, Texas; South Texas Veterans Health Care System, San Antonio, Texas; South Texas Veterans Health Care System, San Antonio, Texas; Cheyenne VA Health Care System, Cheyenne, Wyoming; South Texas Veterans Affairs, San Antonio, Texas; South Texas Veterans Affairs, San Antonio, Texas

## Abstract

**Background:**

Healthcare-associated infections (HAI) have tremendously increased since the coronavirus-19 pandemic. With this increase, various attempts should be made to keep multidrug-resistant organisms in check. In 2021, the Veterans Affairs in San Antonio identified a patient with a multidrug-resistant *Acinetobacter baumannii complex* (MDR-A). This study was undertaken to determine the temporal and spatial relationship of similarly identified MDR-A from other patients at our facility to help prevent spread. We highlight the role whole-genome sequencing plays in a nosocomial outbreak investigation.

**Methods:**

Since the identification of the first MDR-A, we performed interventions to include closure of the unit for new admissions, terminal cleaning, and collection of nasopharyngeal, peri-rectal, urine and wound swabs for surveillance. 15 positive samples were sent from our facility to Multidrug- Resistant Organism Repository and Surveillance Network (MRSN) for genotyping.

**Results:**

Genotyping results confirmed that there were only one to four Single Nucleotide Polymorphism (SNP) variations between three of the patient isolates indicating that these were genetically related with likely nosocomial transmission. All isolates carried an OXA-23 carbapenemase and a 16S rRNA methyltransferase which conferred resistance to all clinically relevant aminoglycosides. Overall, twelve isolates were identified from our facility with < =4 SNP variations interlinking them with prior isolates sent to MRSN. MRSN identified 2-45 SNP variations between 87 isolates from seven different hospitals in South Texas (Figure 1). Environmental sampling in our hospital revealed an *Acinetobacter baumannii* isolated from shower trolleys. Since the organism isolated was identical, spread was presumed secondary to the contaminated shower trolley.

MDR-A cluster indicating interrelation between various isolates from seven different facilities in South Texas.

**Figure:**
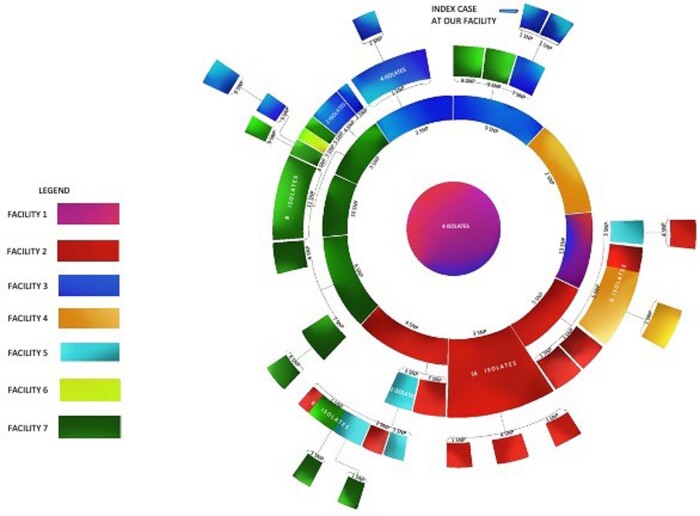

This figure depicts four common isolates pictured in the center, which resulted in SNP variations and spread across seven facilities in South Texas. The SNP variations between isolates are noted here. Several inter-facility spread of MDR-A took place as can be seen here. Abbreviations: SNP, Single Nucleotide Polymorphisms; MDR-A, Multidrug Resistant Acinetobacter baumannii complex

**Conclusion:**

Spread of MDR-A from our index case was linked to transmission from shower trolleys. These were disinfected, and focused disinfection of all high-touch surfaces was undertaken in addition. Further spread was successfully prevented and no other cases with MDR-A identified thereafter. This case highlights the critical steps in outbreak investigation and highlights the importance of whole-genome sequencing in this process.

**Disclosures:**

**All Authors**: No reported disclosures

